# Autoimmuno-Anti-Tumor Immunity – Understanding the Immune Responses against “self” and “Altered-Self”

**DOI:** 10.3389/fimmu.2014.00582

**Published:** 2014-11-14

**Authors:** Fang-Ping Huang

**Affiliations:** ^1^Imperial College London, London, UK; ^2^University of Hong Kong, Hong Kong, China

**Keywords:** autoimmunity, cancer, inflammation, tumorigenesis, immune regulation, immunosuppression, anti-tumor immunity, tumor immunotherapy

The brief description of tumors being “wounds that do not heal” by Dr. Harold F. Dworak nearly three decades ago (*N Engl J Med*, 1986) (1) has provided not only a vivid illustration of neoplastic diseases in general but also, in retrospect conceptually, a plausible immunological definition of cancers. Based on our current understanding in the field, it could have even a multi-dimensional meaning attached with. This relates to several important issues, which need to be addressed further, i.e., in terms of a close link between chronic inflammation and tumorigenesis widely observed; clinical and experimental evidence of immunity against tumors versus the highly immunosuppressive tumor microenvironment being associated; and their underlying immunological mechanisms, oncogenic basis, as well as the true causal relationship in question (2–5).

Recent findings from studies into the pathogenesis of autoimmunity and, more importantly, the mechanisms, which protect against it, have offered some new insights for our understanding in this direction. Chronic or persistent autoimmune-like inflammatory conditions are evidently associated with tumor development. The important question is about their true causal relationship. Chronic or persistent inflammation has been shown to contribute directly to tumor development by triggering neoplastic transformation and production of inflammatory mediators, which could promote cancer cell survival, proliferation, and invasion (2, 3). On the other hand, tumors are mutated self-tissue cells to which the host immune system is largely tolerized otherwise. Although the mutations may give rise to the expression of tumor-specific antigens (TSA) or tumor-associated antigens (TAA), most of these TSAs/TAAs are found to be poor immunogens (6). The ongoing inflammatory conditions may therefore reflect a desperate attempt of the host immune system to mount anti-tumor responses, though ineffectively, being a consequence of the continuous yet largely futile triggering by those poorly immunogenic TSAs/TAAs. Furthermore, during autoimmune or overtly persistent immunological responses, many regulatory mechanisms are triggered in the host in attempts to limit the ongoing harmful inflammatory reactions. Such a negative feedback regulation is known to be crucial in preventing normal individuals from immune-mediated diseases (7). As a result of the negative feedback loop, however, an excessive production of anti-inflammatory or immunosuppressive molecules followed by the exhaustion of the immune effector cells may instead lower the ability of the host immune system to mount specific anti-tumor responses, allowing the escape of tumor or mutated cells from immunosurveillance. This may also help to explain why the most effective way to enhance host immunity against cancer is by targeting the negative arm of immune regulation (8–10).

In this Frontiers Research Topic, we have gathered current views and cutting-edge findings from many experts in these inherent overlapping fields of oncology, autoimmunity, and tumor immunology. It compiles a total of 15 articles in different formats, of concise but informative Mini-review/Reviews, Original Research Articles with novel experimental findings, and some very thought-provoking new Hypothesis/Theory/Opinion/Perspectives. These are now made freely available to our potential readership who may be particularly interested in this cutting-edge area, covering three key issues as outlined below:

- Cancers, Inflammation, and the causal relationship;
- Immune effector and regulatory mechanisms involved in autoimmuno-anti-tumor immunity (AATI);
- Guiding the misguided: AATI alternatively switched on for effective cancer treatment.

For the highly cross-disciplinary nature of this Research Topic, however, the above are reflected in different ways in these articles, crossing throughout the topic. It starts by outlining evidence of the host immune system that may naturally protect against cancers, while it could also cause autoimmunity – being an evolutionally acceptable “side effect” (Chapters 1–2); followed by explaining how autoimmunity could be a “Double-Agent” involved in both tumor-killing and cancer promotion linked to inflammation (Chapters 3–5). It addresses further by dissecting the detailed cellular and molecular mechanisms potentially involved in these processes (Chapters 6–10). These together may help to provide a good basis for the development of novel therapeutic approaches, including stem cell-based immunotherapy, for future cancer treatment (Chapters 11–15). By understanding how the immune system is normally regulated, why dysregulation of which may cause the immunological–oncological related diseases, we aim and hope that the contents of this Research Topic can also trigger further active discussions among scientists in the fields, as to how the so-called “self-reactivity” (autoimmune responses) can be alternatively switched on and redirected, immunologically or molecularly, for effective cancer treatment.

Finally, I would like to thank all the authors for their valuable contributions to this Research Topic, and to express my great appreciations to many members of the journal editorial team, especially Ms. Rosa Mancebo and Ms. Jessica Kandlbauer, for their professional dedication and kind help throughout the process.

## Conflict of Interest Statement

The author declares that the research was conducted in the absence of any commercial or financial relationships that could be construed as a potential conflict of interest.
